# Manufacturing and Characterization of Ti6Al4V Lattice Components Manufactured by Selective Laser Melting

**DOI:** 10.3390/ma7064803

**Published:** 2014-06-23

**Authors:** Sabina L. Campanelli, Nicola Contuzzi, Antonio D. Ludovico, Fabrizia Caiazzo, Francesco Cardaropoli, Vincenzo Sergi

**Affiliations:** 1 Dip. di Meccanica, Matematica e Management—Politecnico di Bari, Viale Japigia 182, 70126 Bari, Italy; E-Mails: nicola.contuzzi@poliba.it (N.C.); antoniodomenico.ludovico@poliba.it (A.D.L.); 2 Dip. di Ingegneria Industriale—Università degli Studi di Salerno, Via Giovanni Paolo II 132, 84084 Fisciano (SA), Italy; E-Mails: f.caiazzo@unisa.it (Fa.C.); fcardaro@unisa.it (Fr.C.); sergi@unisa.it (V.S.)

**Keywords:** Ti6Al4V titanium alloy, selective laser melting, micro-lattice structures, tailored porosity, characterization

## Abstract

The paper investigates the fabrication of Selective Laser Melting (SLM) titanium alloy Ti6Al4V micro-lattice structures for the production of lightweight components. Specifically, the pillar textile unit cell is used as base lattice structure and alternative lattice topologies including reinforcing vertical bars are also considered. Detailed characterizations of dimensional accuracy, surface roughness, and micro-hardness are performed. In addition, compression tests are carried out in order to evaluate the mechanical strength and the energy absorbed per unit mass of the lattice truss specimens made by SLM. The built structures have a relative density ranging between 0.2234 and 0.5822. An optimization procedure is implemented via the method of Taguchi to identify the optimal geometric configuration which maximizes peak strength and energy absorbed per unit mass.

## 1. Introduction

Selective Laser Melting (SLM) is a process which belongs to Rapid Manufacturing (RM), which directly produces end-use products or parts. The RM is a direction of Rapid Prototyping (RP) technology, other than Rapid Tooling (RT). SLM represents an evolution of the Selective Laser Sintering (SLS) process that was developed and patented by Carl Deckard and Joe Beaman at the University of Texas at Austin in the mid-1980s for producing plastic prototypes [1,2]. The success of SLM, which is probably the most rapidly growing technique in RP and Layer manufacturing (LM) technologies, results mainly from the ability to create metal parts with complex shapes and intrinsic engineered features. Moreover, this technique is particularly interesting for the possibility of producing parts with mechanical properties better or at least comparable with those of components produced with traditional processes. This result can be demonstrated by several studies. Ng *et al.* [3] investigated the effects of laser processing parameters on the microstructure and mechanical properties of selective laser-melted magnesium finding that hardness values and elastic modulus of the laser melted magnesium were comparable with the cast magnesium ingot. Attar *et al.* [4] reported that SLM was able to produce high-strength CP-Ti parts superior to those of conventional processes. Prashanth *et al.* [5] built Al–12Si samples characterised by a maximum strength two times higher than the corresponding values of the cast material.

Most SLM literature concerns the optimization process parameters in order to obtain almost full density of parts and good mechanical properties of the bulk materials [6,7,8,9].

In recent years, SLM has been used to fabricate lattice structures for the production of lightweight components, because of the high geometrical freedom that can be realized in comparison to conventional manufacturing processes [10,11,12,13,14].

The interest in manufacturing parts with tailored porosity is rapidly growing in scientific research. This aspect is also connected to the opportunity of employing innovative technologies based on additive techniques of Layer Manufacturing (LM). There are several applications of porous materials ranging from the biomedical to aerospace sectors, where they are used in heat transfer and acoustics as filters [15,16].

The potential of the SLM Ti6Al4V material as high performance parts in aerospace, automotive and medical applications is actively being explored by researchers and companies throughout the world. Studies on SLM Ti6Al4V micro-lattice structure as core material in sandwich construction showed that this material has significant potential that merits further examination and analysis [17,18].

Other researchers have shown the great potential of Ti6Al4V alloy in future aerospace applications [19], or in the civil aircraft field [20]. Further studies have shown the possibility of using this titanium alloy for biomedical applications [21].

Particular attention is focused on the production of biomedical customised net-shape prostheses or devices from metallic powders. Implants are traditionally manufactured via investment casting, forging or machining, and formed by different components with bulk properties that are optimized for particular design criteria, such as bio-compatibility, strength, flexibility, wear resistance or bone ingrowth. These parts are bonded or mechanically attached together. One challenge to this traditional method is achieving sufficiently strong, permanent bonds between the implant sections that will not fail under the fatigue loading imposed by the body of the patient.

An approach to avoid joining mismatches is the use of monolithic metallic implants, but the latter solution is characterized by two types of issues: from a mechanical point of view these devices present dissimilar properties compared with bone ones, causing the stress shielding phenomenon with a consequent decay of osseous tissue and a reduction in the *in vivo* duration of the implants; considering the biological aspect, the critical point is osteointegration, as traditional prostheses have to be further treated to obtain surfaces which facilitate the interaction of biological fluids and consequently strengthen the bond between tissue and implant, accelerating the healing process and the vascularisation. In order to solve the last issue, coatings with hydroxyapatite, PMMA (Polymethylmethacrylate) or surface treatments are usually employed, but these processes lengthen the production cycle [22,23].

SLM represents an alternative to traditional processes [24,25,26]; the advantage of such technology is connected with the possibility of manufacturing complex geometries directly from a three-dimensional CAD model, obtaining parts with tailored, interconnected porosity and surface features which allow biological processes and bone ingrowth. A further positive aspect is represented by the reduction of production time compared to traditional techniques. Indeed, data obtained from CT scans are used to design an optimal fit prosthesis via CAD/CAM. This model can be adopted to produce a customised implant through layer fabrication.

In order to create a metallic implant with mechanical properties similar to bone ones, a tailored porosity in the component is necessary. The presence of cavities reduces stiffness mismatch and, as a consequence, stress shielding [27]. A porous part can be produced via LM in two different ways: The first approach considers the bulk model and creates internal pores with powder partial fusion through the adequate choice of process parameters [27,28]; the latter uses a model with cavities and characterized by periodic cellular unit [24,29,30,31]. A third approach combines both above-quoted methods [32].

The first method does not allow reaching porosity greater than a certain percentage, since the connection among the powder particles is not feasible with too low values of specific energy [33]. Furthermore, a critical aspect is the removal of loose particles, which may cause human irritations in biomedical devices. The second approach presents minor restrictions.

In this paper, the feasibility of manufacturing lattice structures with tailored porosity adopting SLM process and their properties was studied. A Ti6Al4V powder, biocompatible and corrosion resistant, is used employing an EOSINT M270 titanium version laser sintering system with optimized exposure parameters to obtain full density of laser sintered parts. The lattice structure considered in the experimental phase is a pillar textile one, comprised of four vertical strut columns and four couples of struts inclined at ±45° with respect to cell axes of symmetry. A previous work on this structure was performed by Contuzzi *et al.* [15] processing via SLM the 18 Ni Marage 300 steel. They demonstrated that using vertical reinforcements loading capability of the base structure could be significantly improved.

Different topology structures were studied considering three parameters: the size cell (*L*); the truss size (*t*); and the number of reinforcements (*R*). In order to reduce the number of experiments a Taguchi experimental plane was used. The performance of these structures were analysed in terms of dimensional accuracy, roughness, micro-hardness, mechanical strength under compression and energy absorbed per unit mass.

## 2. Results and Discussion

### 2.1. Visual Inspections

**Figure 1:**
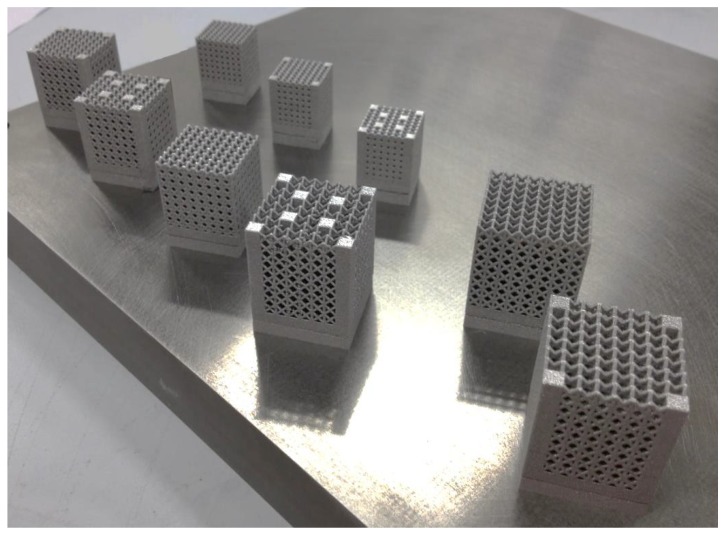
Samples on the building platform.

Figure 1 shows the samples on the building platform. Before the part removal from the platform, a heat treatment was carried out at 650 °C for 2 h in argon atmosphere in order to avoid oxidation. The aim of this procedure is to operate a relief of stresses due to high thermal gradients experienced during the manufacturing process. Then, specimens were sandblasted to remove partially molten particles.

**Figure 2:**
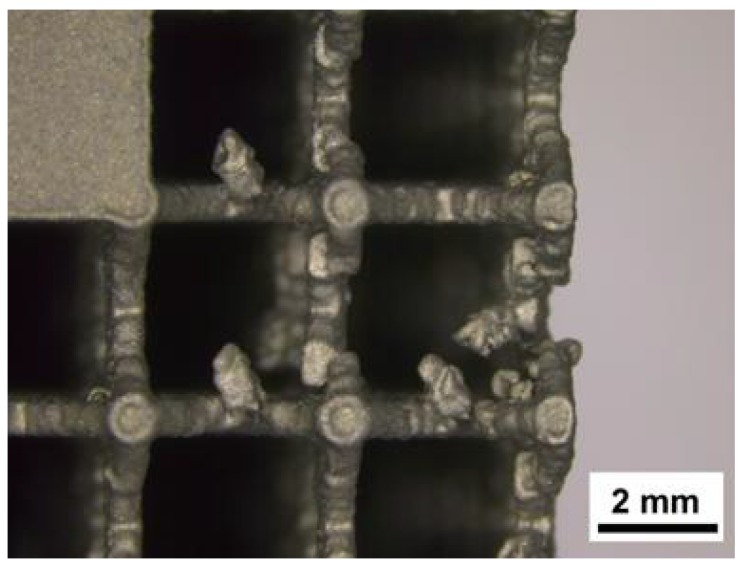
Defects observed in test 7.

The only non-stochastic configuration which created issues during the fabrication is corresponding to sample 7. Observing the defects, it is noticed that some trusses have been bent in the recoating phase. This phenomenon is connected both with truss and cell size; indeed, the coupling of minimum thickness of the truss with the maximum cell size resulted in the instability of laser sintered part (Figure 2). The other samples were not characterized by any imperfections proving the capability of the SLM technique.

### 2.2. Dimensional Analysis of Lattice Structures and Cell Features

A CMM was employed to perform metrological analysis and verify accuracy of the manufacturing process. In particular, lattice structure sizes were measured and their average values were reported in Table 1, where *a*_eff_ is the measure of the effective side of the structure; and Δ the error in µm. The sample height was not considered since it is affected more by the finishing operations than the building capabilities of SLM. It can be inferred that deviations from model dimensions are quite small; even considering each measurement, the gap is less than 50 µm.

**Table 1:** Results of the metrological analysis.

Test	*a* (mm)	*a*_eff_ (mm)	Δ (µm)
1	16.5	16.477	−23
2	16.6	16.578	−22
3	16.7	16.663	−37
4	20.5	20.531	30
5	20.6	20.590	−10
6	20.7	20.681	−19
7	24.5	24.491	−9
8	24.6	24.614	14
9	24.7	24.705	5

**Figure 3:**
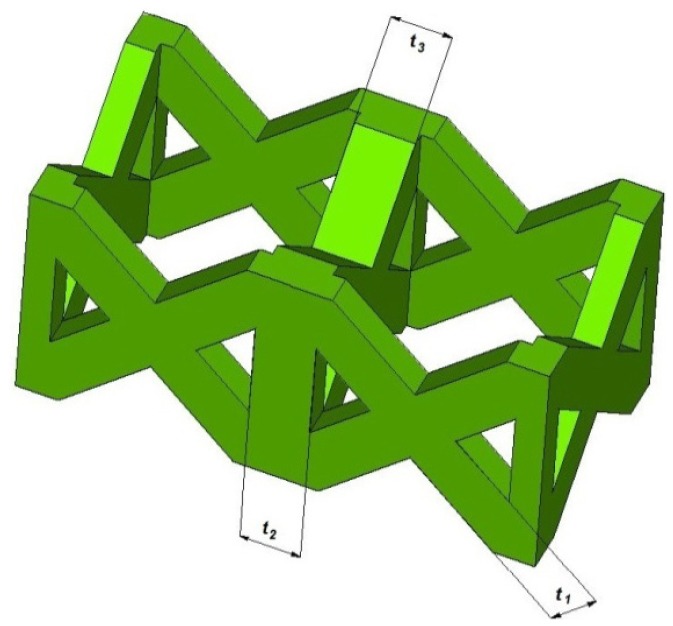
Truss thickness measured via stereomicroscope.

The layer fabrication of the process leads to a certain dimensional variation at the level of the single cell. In particular, trusses shown in Figure 3 should present all the same thickness. Table 2 reports the average values observed for trusses through the macrographs acquired via a Leica S8AP0 stereomicroscope (Leica, Solms, Germany) and elaborated with Leica Application Suite software. A deviation from the theoretical dimensions is highlighted, in conjunction with a discrepancy between trusses with variable orientation.

This anomaly also affects the effective relative density (ρ_reff_) of the lattice structures in comparison with designed porosity (ρ_r_). ρ_r_ is evaluated as the ratio between volume of CAD model (*V*_t_) and volume of full element (*V*_b_); whereas, effective volume of the lattice structure (*V*_eff_) is calculated through cell feature measurements.

**Table 2:** Results of stereomicroscope analysis and evaluation of effective relative density.

Test	*t* (μm)	*t*_1_ (μm)	*t*_2_ (μm)	*t*_3_ (μm)	*V*_b_ (mm^3^)	*V*_t_ (mm^3^)	ρ_r_	*V*_eff_ (mm^3^)	ρ_reff_
1	500	515	523	508	4356	1389	0.3190	1441	0.3308
2	600	626	609	621	4409	2026	0.4596	2102	0.4768
3	700	725	716	703	4462	2598	0.5822	2635	0.5905
4	500	513	506	507	8405	2354	0.2800	2401	0.2857
5	600	614	610	608	8487	3414	0.4022	3467	0.4085
6	700	708	708	700	8570	3239	0.3779	3265	0.3810
7	500	512	512	510	14406	4108	0.2852	4178	0.2900
8	600	615	604	604	14524	3245	0.2234	3319	0.2285
9	700	715	724	710	14642	4962	0.3388	5072	0.3464

The difference in terms of relative density, however, is quite limited and presents a maximum value of 1.7%. This value is acceptable when compared with the deviation obtained by Lin *et al.* [30], using the same process and powder, which reaches 7%. Moreover, differences in relative density even more marked, over 20%, have been observed in the experimentation of Parthasarathy *et al.* [24], who works with the same alloy but adopts the technique of EBM, demonstrating a less reliable reproduction of the CAD model.

### 2.3. Roughness, Micro-Hardness and Microstructure

Undesirable in other sectors, in medical implants a coarse surface is an advantage because it enhances bone-implant fixing and accelerates the healing process as roughness increases osteo-conductivity.

The bond between the implant and bone tissue is excellent for *R_a_* values ranging between 1 and 10 µm [34]. After performing a preliminary test on specimen roughness, a cut-off of 2.5 mm was chosen according to ISO 4288–2000 standard [35] to guarantee a good reliability in *R_a_* range for samples under examination.

*R*_a_ roughness for lattice structures ranges from 6 to 14 µm and it is a function of the sandblasting process. Indeed, since the sandblasting is manual, sides directly exposed to the sand jet present a low irregularity. As a consequence, carrying out a homogeneous treatment on every surface, adequate roughness of laser sintered parts may be obtained.

**Figure 4:**
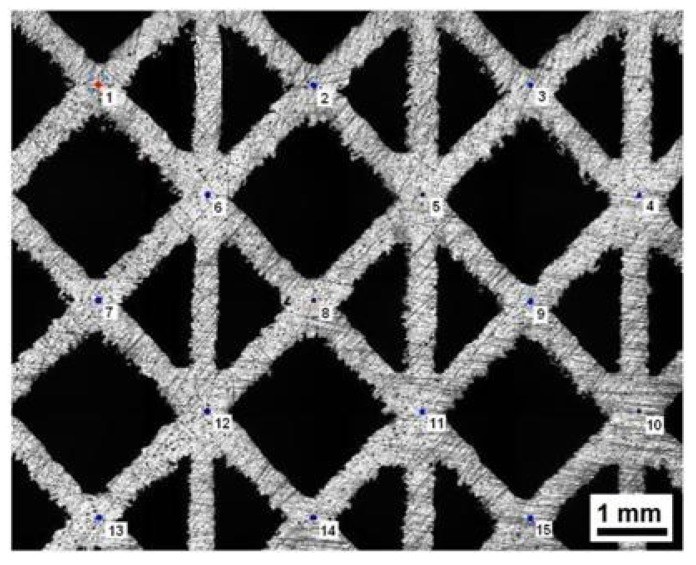
Truss thickness measured via stereomicroscope.

Micro-hardness tests were performed on grinded surfaces of different samples. As an example, results are shown for specimen in condition 4 (Table 3). The indentations follow the path pointed out in Figure 4. Micro-hardness is 398 ± 11 HV_0.3_, which is greater than the minimum value of titanium alloys for medical use (349 HV) [36].

The microstructure of the specimens recalls the typical one obtained for a sample in Ti6Al4V quenched from a temperature above the beta transus (about 995 °C). The behaviour during solidification is due to the fact that titanium alloys have different allotropic forms as a function of temperature. In particular, above the so-called beta transus temperature, the microstructure changes from alpha (hexagonal close packed) to beta (body centred cubic). Ti6Al4V is an alpha + beta alloy, because it contains Al which stabilizes the alpha phase, and V as a beta stabilizer. At room temperature equilibrium, this alloy supports a mixture of alpha and beta phases.

**Table 3:** Results of micro-hardness for specimen in condition 4.

Indentation	HV_0.3_	*d*_1_ [µm]	*d*_2_ [µm]
1	415	36.5	36.8
2	405	37.3	36.8
3	412	36.9	36.6
4	397	37.5	37.3
5	395	37.5	37.5
6	401	38.0	36.5
7	379	38.3	38.4
8	386	37.7	38.3
9	390	37.8	37.8
10	417	36.1	36.9
11	400	36.9	37.7
12	381	37.4	39.0
13	403	37.4	36.9
14	390	37.9	37.7
15	397	37.9	36.9

When a specimen is quenched from a temperature above the beta transus, martensitic alpha is obtained from beta grains. In alloys with low concentrations of beta-stabilizing elements, like Ti6Al4V, the martensite has a distorted hexagonal crystal lattice [28]. Rapid solidification after the interaction with the laser beam leads to a greater hardness value compared to those ones of bulk base material, as showed by previous micro-hardness tests. Furthermore, no peculiar grain orientation was observed proving the SLM’s capability to produce parts with isotropic properties.

### 2.4. Compression Tests

**Figure 5:**
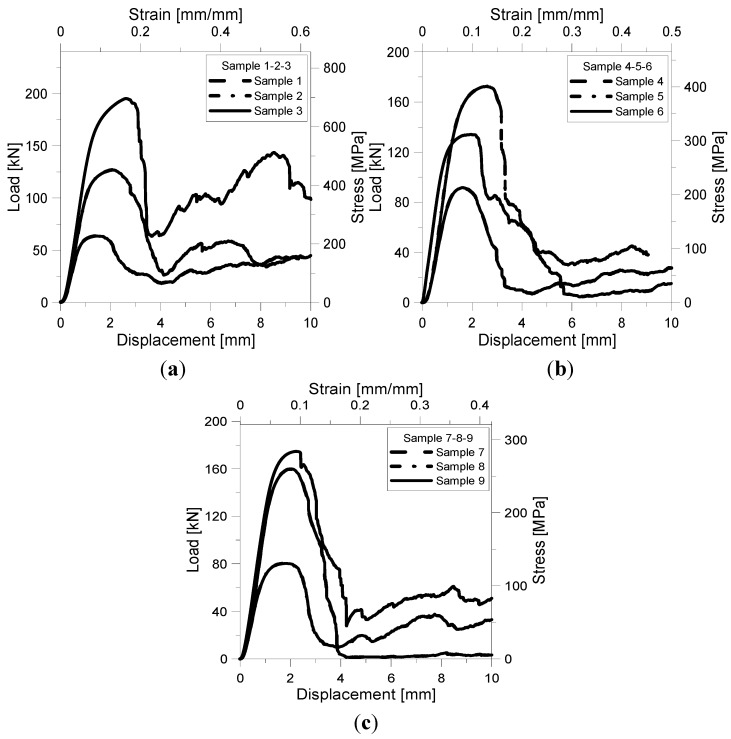
Load-displacement/stress strain curves for *L* = 2 mm sample (**a**); *L* = 2.5 mm sample (**b**); and *L* = 3 mm sample(**c**).

Figure 5a–c shows the load-displacement/stress-strain curves for cell size *L* = 2, 2.5, 3 mm samples. Stress was determined as the ratio between the applied load measured by the load cell and the nominal cross-section of the specimen; strain was evaluated as the ratio between the measured shortening and nominal height of the specimen. Values of stress and strain were derived from the load-displacement curves recorded experimentally. Samples exhibit similar compressive stress-strain behaviour. After an initial region of linear elasticity, a gradual yield occurs followed by a peak compressive strength (σ*_pk_*). This strength corresponds at the maximum stress that the structure can support before collapse. Continued loading resulted in a plastic collapse until breaking and densification. The degree of softening seems to be more affected by cell size and size of strut edge than by number of reinforcements. For impact energy absorption applications, a stress *versus* strain response with little or no softening after yield is desirable [37,38]. Samples without reinforcements (1, 6 and 8) exhibit this behaviour having the smaller softening after yield. On the other hand, samples 2, 3 and 9 are able to bear load again and, therefore, could still absorb energy under compressive loading.

Values of peak and collapse strength (σ_sf_) are listed in Table 4. The collapse strength was calculated as the stress reached by the structure during the plastic collapse soon after the maximum strength. The maximum load carrying capability is achieved by sample 3 which is characterised by the smaller cell size, the highest size of strut edge and the highest relative density (ρ_r_ = 0.5822).

**Table 4:** Values of peak and collapse strength for the samples.

Sample	σ_pk_ (MPa)	σ_sf_ (MPa)	ρ_r_
1	232.5	76	0.3190
2	456.6	111	0.4596
3	692.3	247	0.5822
4	215.8	40	0.2800
5	404.2	20	0.4022
6	309.8	78	0.3779
7	265.9	5.32	0.2852
8	131.6	18	0.2234
9	283.5	58	0.3388

**Figure 6:**
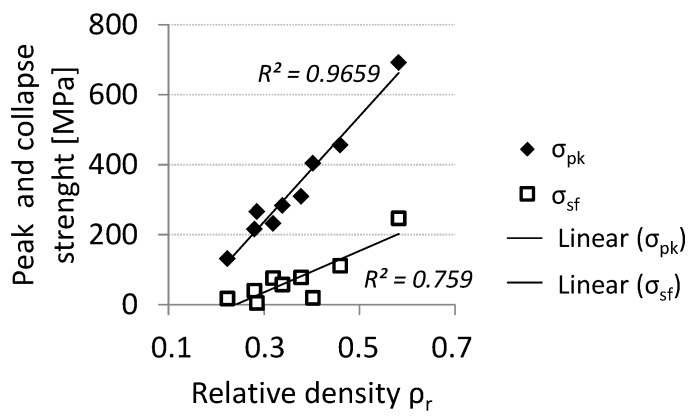
Peak and collapse compressive strength *vs.* relative density.

Thus, the introduction of vertical reinforcements improves the load carrying capability of the lattice truss, as expected and as already demonstrated by Contuzzi *et al.* [15] with similar structures built by SLM using a maraging steel powder. Sample 3 also exhibits the lower degree of softening having the maximum collapse strength. Peak and collapse stress values increase almost linearly with relative density (Figure 6): The correlation coefficient for the linear fitting is 0.965 for σ_pk_ and 0.759 for σ_sf_.

**Figure 7:**
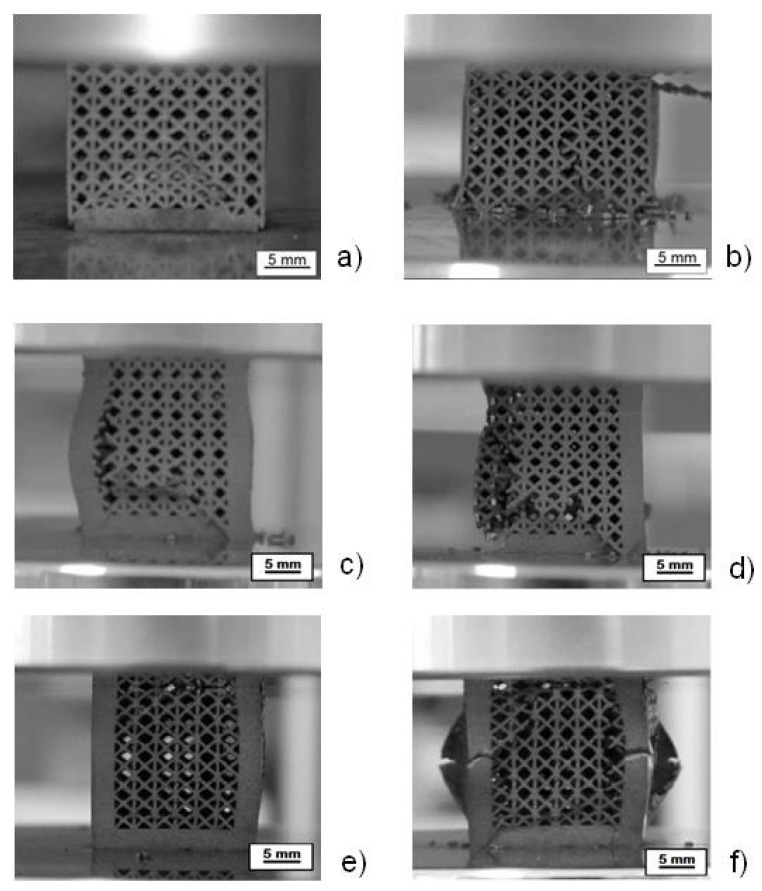
Deformed structure without reinforcements (sample 6) (**a**,**b**); with four reinforcements (sample 9) (**c**,**d**); and with eight reinforcements (sample 7) (**e**,**f**).

Figure 7 shows the collapse mode for samples 6, 9 and 7, which are representative of the three kinds of structures. Specifically, Figure 7a,b illustrates the deformation of the base pillar textile lattice structure without reinforcements. Struts, after buckling, experience first a plastic collapse (a) and later on the rupture of structure (b). The structure with four reinforcements (c and d) breaks and collapses after buckling of vertical reinforcements. The structure with eight reinforcements (e and f) experiences first buckling and then breaking of the vertical reinforcements, followed by the collapse of the entire structure.

### 2.5. Statistical Analysis

In order to investigate the influence of the considered factors on the compressive behaviour of samples, the Analysis of Variance (ANOVA) was performed. The investigated factors are the cell size, the truss size and the number of vertical reinforcements. The analyzed output factors are: peak strength, collapse strength and the energy absorbed per unit mass.

The merits of different materials for impact energy absorption can be compared by determining the strain energy absorbed during their compression up to densification [38]. The energy absorbed per unit volume *W_v_*, [39] is defined by Equation (1):

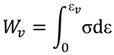
(1)


In this case, the energy was calculated till a strain of 0.4 was reached. Energy absorbers of minimum mass are also important for weight sensitive applications. The energy absorbed per unit mass is defined by Equation (2):

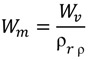
(2)
where ρ is the density of the parent alloy.

The results of ANOVA are summarized in Table 5, Table 6 and Table 7. The *p*-value tells whether the effect for that term is significant. If the effect of a discrete factor is significant, then the variance of the factor is not zero. If a calculated p-value is greater than the level of significance, the effect of the parameter is judged not to be significant. The level of significance set to 0.07 allows selecting the parameters whose effect is not negligible from a statistical point of view [40]. The ANOVA showed that only peak strength and energy absorbed per unit mass are affected by the three factors *L*, *t* and *R*, while collapse strength is not influenced by the variation of these factors.

**Table 5:** Analysis of variance for peak strength.

Factors	Seq SS	Adj SS	Adj MS	F	*p*-Value
*L*	84,056	84,056	42,028	28.08	0.034
*t*	54,451	54,451	27,226	18.19	0.052
*R*	79,861	79,861	39,931	26.68	0.036

**Table 6:** Analysis of variance for collapse strength.

Factors	Seq SS	Adj SS	Adj MS	F	*p*-Value
*L*	23,912	23,912	11,956	5.87	0.146
*t*	13,778	13,778	6,889	3.38	0.228
*R*	1,716	1,716	858	0.42	0.704

**Table 7:** Analysis of variance for energy absorbed per unit mass.

Factors	Seq SS	Adj SS	Adj MS	F	*p*-Value
*L*	0.089387	0.089387	0.044694	111.30	0.009
*t*	0.041269	0.041269	0.020635	51.39	0.019
*R*	0.011188	0.011188	0.005594	13.93	0.067

An optimization procedure was performed by means of the method of Taguchi. A loss function was defined which measured the deviation of the quality characteristic from a desired value. In order to assess the influence of each input factor on the output, the means and Signal-to-Noise ratios (S/N), for each control factor, were calculated. The signals are indicators of the effect on the average responses and the noises are measures of the influence on the deviations from the average responses, which accounts for the sensitiveness of the experiment output to noise factors. The appropriate S/N ratio must be chosen using previous knowledge, expertise, and understanding of the process. There are three categories of the quality characteristic in the analysis of the S/N ratio, *i.e.*, the lower-the-better, the larger-the-better, and the nominal-the-better. In this study, the goals are to identify the factors which maximize peak strength and the energy absorbed per unit mass *W_m_* and which are robust to noises.

**Figure 8:**
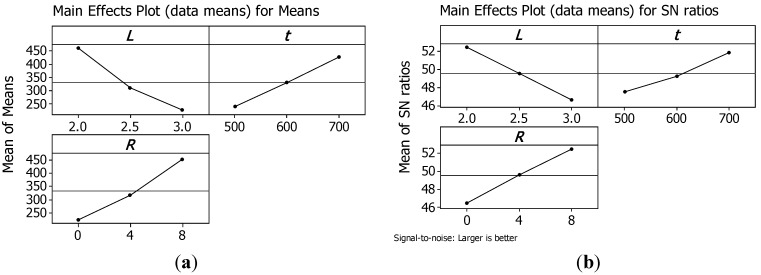
Main effects plot for means (**a**) and for S/N ratios (**b**) for peak strength.

**Figure 9:**
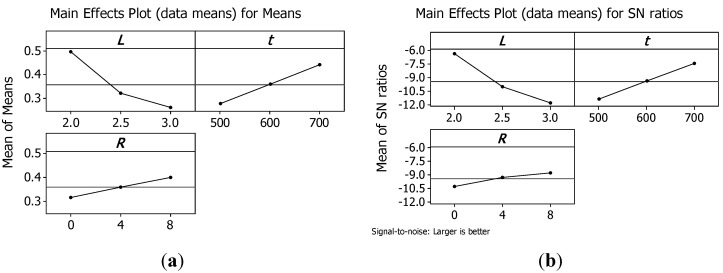
Main effects plot for means (**a**) and for S/N ratios (**b**) for energy absorbed per unit mass *W_m_*.

Therefore, in this study, the S/N ratio was chosen according to the criterion the-larger-the-better. The S/N ratio for each level of process parameters is computed based on the S/N analysis. Regardless of the category of the quality characteristic, a larger S/N ratio corresponds to a better quality characteristic. Therefore, the optimal level of the process parameters is the level with the highest S/N ratio. Figure 8 and Figure 9 show the main effect plots for means and for S/N ratios, respectively, for peak strength and for the energy absorbed per unit mass *W_m_*.

Analysing plots, it is evident that both σ_pk_ and *W*_m_ can be maximised using structures with the minimum cell size *L*; the maximum truss size *t*; and the maximum number or reinforcements *R*.

Thus, the structure that presents the maximum load capability has also the maximum energy absorbed per unit mass till a strain of 0.4. Hence, the best configuration is reached by sample 3 that has a relative density of 0.5822.

## 3. Experimental Section

Referring to the choice of geometry, manufacturing technology has two main constraints. The former is connected with the minimum track which the laser beam is able to produce. The threshold value is 200 μm equal to the size of melted zone with a single spot. The latter concerns the lowest angle between the truss and building platform. In literature, some works showed that it is not recommended to use angles less than 45°, in order to not compromise laser sintered part stability [15,41,42]. Indeed, with small angles, the overlap between each layer is minimal and this may cause their removal during the recoating phase. Moreover, previous works demonstrated that the pillar textile concept is one of the best structures in terms of low relative density and large compressive strength [15].

**Figure 10:**
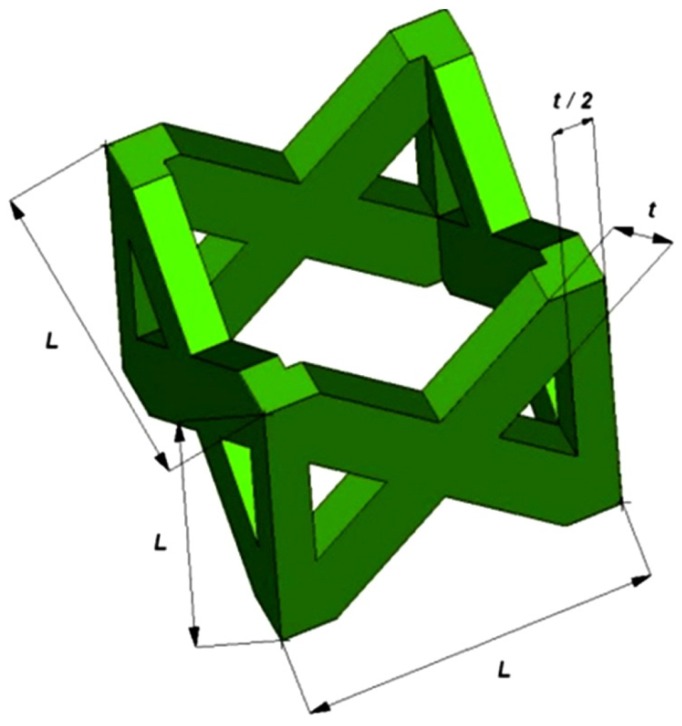
Schematic illustration of the pillar textile unit cell and main geometric dimensions.

The pillar textile lattice structure, comprised of four vertical strut columns and four pairs of struts inclined at ±45° with respect to cell axes of symmetry, has been selected. Figure 10 shows the unit cell of the lattice structure chosen in this study. *L* is the length of the base side of the unit cell (*i.e.*, the cell size) and *t* is the thickness of the single strut edge.

**Figure 11:**
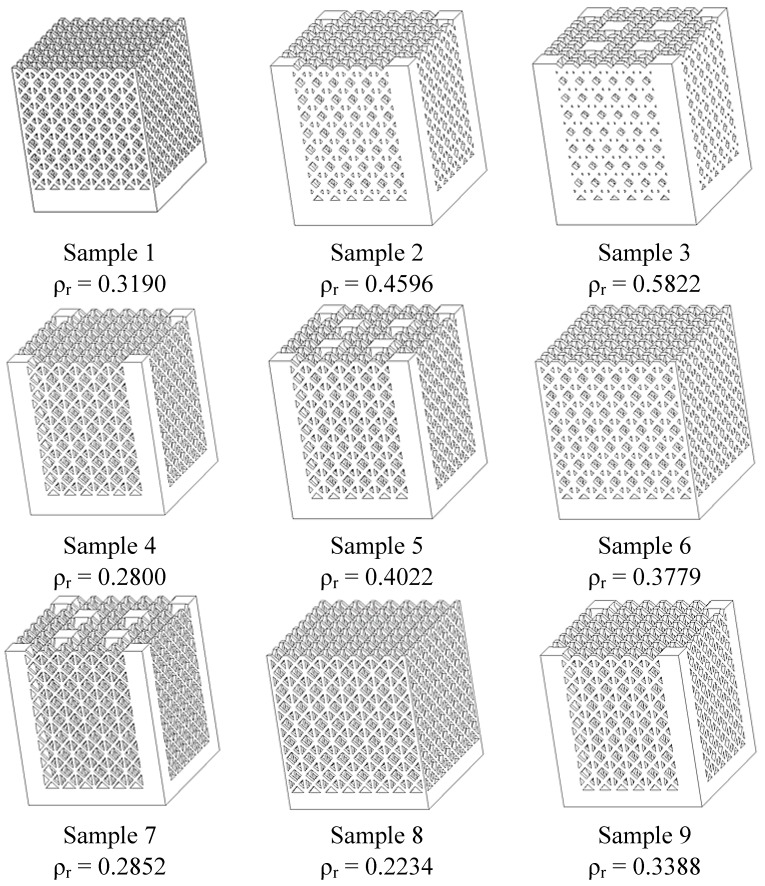
Cell topologies of the nine combinations of parameters.

In order to test the suitability of SLM process in the examined context, an experimental campaign was planned to compare the behaviour of the above structures with variable cell (*L*) and truss size (*t*). Furthermore, vertical bars (*R*) to reinforce the pillar lattice structures and improve stiffness were considered in some topologies. To minimize the number of experiments, Taguchi’s experiment planning was used. The experimental set-up was according to the L9 orthogonal array, as illustrated in Table 8, where *a* is the side and *h* the height of complete lattice structures with a square section. Three factors were considered as input variables of the structures: *L*, *t* and *R*. The three factors were changed on three different levels. Figure 11 shows the nine combinations of samples, which have relative density (ρ*_r_*) ranging between 0.2234 and 0.5822.

**Table 8:** Fractional plan used for the experimental phase.

Test	*L* (mm)	*t* (µm)	*R*	*a* (mm)	*h* (mm)	ρ_r_
1	2.0	500	0	16.5	19.0	0.3190
2	2.0	600	4	16.6	19.0	0.4596
3	2.0	700	8	16.7	19.0	0.5822
4	2.5	500	4	20.5	23.0	0.2800
5	2.5	600	8	20.6	23.0	0.4022
6	2.5	700	0	20.7	23.0	0.3779
7	3.0	500	8	24.5	27.0	0.2852
8	3.0	600	0	24.6	27.0	0.2234
9	3.0	700	4	24.7	27.0	0.3388

Lattice structures were manufactured using an EOSINT M270 titanium version system. Machine specifications are given in Table 9. Ti6Al4V powder with a mean particle size of 30 µm was used in this investigation and alloy chemical composition is listed in Table 10. The powder quality is important to reduce the content of impurities (oxygen, hydrogen and nitrogen), which might negatively affect mechanical properties of laser sintered parts with phenomena like embrittlement. Furthermore, the composition fulfils the requirements of ASTM F1472 [43] regarding maximum concentration of impurities in titanium alloy for surgical implant applications. During the processing phase, the CAD model is sliced into thin layers whose thickness is 30 µm. Within the laser sintering machine, the chamber is filled with argon in order to operate in a controlled atmosphere with percentages of oxygen lower than 0.13% (to prevent oxidation during sintering and explosions because of fumes and vapours). The titanium powder from the dispenser is moved by the recoater and layered on a building platform, where the laser beam selectively melts powder bed following the scan path. After that, the dispenser is lifted up to supply material for a new layer and the process platform is lowered by the thickness of one layer. This cycle is repeated until the parts are completed.

**Table 9:** EOSINT M270 titanium version specifications.

Effective Building Volume	250 × 250 × 215 mm^3^
Building speed	2 ÷ 20 mm^3^/s
Layer thickness	20 ÷ 100 μm
Laser type	Yb–fibre
Maximum power	200 W
Precisions optics	F-theta lens, high speed scanner
Focused spot diameter	0.090 mm

**Table 10:** Chemical composition of Ti6Al4V (wt%), * Max.

Al	V	O	N	C	H	Fe	Ti
5.5 ÷ 6.75	3.5 ÷ 4.5	0.2 *	0.05 *	0.08 *	0.015 *	0.30 *	Bal.

The exposure parameters for manufacturing the samples in Ti6Al4V were chosen in order to obtain full density of parts by SLM, exploiting past experience on the same alloy, and are summed up in Table 11 [28]. These parameters affect energy density, which is a key factor in SLM. Indeed, too high values of energy per volume lead to an excessive melting of the layers with a substantial shrinkage and a consequent balling. This phenomenon occurs when the molten material does not wet the underlying substrate due to the surface tension, which tends to spheroidise the liquid. On the other hand, too low values of energy density are not suitable to ensure adhesion between consecutive layers, because the penetration depth is not adequate [33]. The SLM process is the same as SLS except for the much higher laser energy density required. The amount of energy density causes the powder to fully melt in SLM and to partial melt in SLS. In SLM, nearly full density parts can be produced without the need for post-processing steps [44,45].

**Table 11:** Exposure parameters adopted for manufacturing the samples.

Factor	Symbol	Value
Laser power	*P*	170 W
Scan speed	*v*	1.25 m/s
Hatch spacing	*hs*	0.10 mm
Scan length	*l*	5 mm
Layer thickness	*s*	30 µm

To perform the characterization of the specimens, dimensional measurements were carried out both for single cell and whole structure. The attention was focused on accuracy as past studies pointed out that additive techniques do not allow the faithful replication of the model with significant changes in terms of porosity content and mechanical behaviour [29,30]. The whole samples were examined using a coordinate measuring machine (CMM) DEA Global Image Clima (Hexagon Metrology, Stockholm, Sweden), with a maximum permissible μm error of 1.5 + *M*/333, where *M* is measured in millimetres. In order to perform analysis for cell features, macrographs were acquired via a Leica S8AP0 stereomicroscope and elaborated with Leica Application Suite software.

A further analysis concerns the roughness of laser sintered components since this factor has a high impact on osteointegration and therefore on interactions of implant surfaces with biological fluids [39,46]. Roughness measurements were carried out through a Taylor-Hobson Form Talysurf 50 (Taylor-Hobson, Leicester, England).

Moreover, micro-hardness tests were performed on samples to make a comparison with hardness of Ti6Al4V alloy adopted in the medical field. In the experimental campaign, Vickers micro-hardness was tested according to EN ISO 6507-1 [47] using a ZwickRoell ZHVµ-A instrument (ZwickRoell, Ulm, Germany); a load of 2.94 N (*i.e.*, 0.3 kgf) was used for 10.5 s at 60 µm/s speed.

The nine cell variants considered in this study were all tested under uniaxial compression. As underlined by Harrysson *et al.*, this kind of test is really important for hip stems and, in general, for highly-stressed prostheses [25]. Mechanical tests were performed under displacement control with an Instron 4467 machine equipped with a 200 kN load cell; end-shortening was supplied to the specimen by setting compression speed to 0.5 mm/min.

## 4. Conclusions

In this paper, the feasibility of manufacturing lattice structures with tailored porosity adopting SLM process and their properties has been studied. A Ti6Al4V powder, biocompatible and corrosion resistant, was used employing an EOSINT M270 titanium version laser sintering system with optimized exposure parameters to obtain the full density of laser sintered parts.

The lattice structure considered in the experimental phase is a pillar textile one, comprised of four vertical strut columns and four pairs of struts inclined at ±45° with respect to the cell axes of symmetry. A campaign was planned to compare the behaviour of the above structures with variable cells, truss sizes and vertical bars as reinforcements. To minimize the number of experiments, Taguchi’s experiment planning was used.

The only non-stochastic configuration which created issues during the fabrication is corresponding to lattice structure with minimum thickness of the truss and maximum cell size. The other samples were not characterized by imperfections proving the capability of the SLM technique.

The results of metrological analysis with a CMM demonstrate the great accuracy of the manufacturing process with a gap in model dimensions less than 50 µm. The layer fabrication of the process leads a certain dimensional variation at the level of the single cell, which also affects the effective relative density of the lattice structures in comparison with designed porosity. The difference in terms of relative density, however, is quite limited and acceptable when compared with the deviation obtained in other experimentations, using a similar process and powder.

From the results of the roughness test, it is inferred that adequate roughness of laser sintered parts may be obtained with sandblasting to enhance bone-implant fixing and accelerate the healing process. Regarding micro-hardness tests, the average value is greater than the minimum one of titanium alloys for medical use (349 HV). Indeed, the high energy density of laser beams causes the formation of martensitic alpha, a microstructure which is characterised by great resistance.

Compression tests were performed in order to evaluate the mechanical behaviour under compression of the micro-lattice topology variants. It is found that the load carrying capability of the structure is affected by the number of vertical reinforcements, by strut edge size and cell size. Peak and collapse stress values increase almost linearly with relative density. The maximum load carrying capability and the lower degree softening is achieved by sample 3 which is characterised by the smaller cell size, the highest size of strut edge and the highest relative density. This result is also demonstrated by statistical optimisation performed by the method of Taguchi. This analysis has shown that the structure that presents the maximum load capability has also the maximum energy absorbed per unit mass.

## References

[1] Schleifenbaum H., Diatlov A., Hinke C., Bültmannn J., Voswinckel H. (2011). Direct photonic production: Towards high speed additive manufacturing of individual goods. Prod. Eng. Res. Dev..

[2] Schleifenbaum H., Meiners W., Wissenbach K., Hinke C. (2010). Individualized production by means of high power selective laser melting. CIRP J. Manuf. Sci. Technol..

[3] Ng C.C., Savalani M.M., Lau M.L., Man H.C. (2011). Microstructure and mechanical properties of selective laser melted magnesium. Appl. Surf. Sci..

[4] Attar H., Calin M., Zhang L.C., Scudino S., Eckert J. (2014). Manufacture by selective laser melting and mechanical behaviour of commercially pure titanium. Mater. Sci. Eng. A.

[5] Prashanth K.G., Scudino S., Klauss H.J., Surreddi K.B., Löber L., Wang Z., Chaubey A.K., Kühn U., Eckert J. (2014). Microstructure and mechanical properties of Al-12Si produced by selective laser melting: Effect of heat treatment. Mater. Sci. Eng. A.

[6] Casavola C., Campanelli S.L., Pappalettere C. (2009). Preliminary investigation on the residual strain distribution due to the Selective Laser Melting Process. J. Strain Anal..

[7] Campanelli S.L., Contuzzi N., Angelastro A., Ludovico A.D. (2010). Capabilities and performances of the Selective Laser Melting process. New Trends in Technologies: Devices, Computer, Communication and Industrial Systems.

[8] Kruth J.P., Mercelis P., Van Vaerenbergh J., Froyen L., Rombouts M. (2004). Selective laser melting of iron-based powder. J. Mater. Process. Technol..

[9] Campanelli S.L., Contuzzi N., Ludovico A.D. (2010). Manufacturing of 18 Ni Marage 300 steel samples by selective laser melting. Adv. Mater. Res..

[10] Yadroitsev I., Shishkovsky I., Bertrand P., Smurov I. (2009). Manufacturing of fine-structured 3D porous filter elements by selective laser melting. Appl. Surf. Sci..

[11] Reinhart G., Teufelhart S. (2012). Load-adapted design of generative manufactured lattice structures. Phys. Procedia.

[12] Smith M., Cantwell W.J., Guan Z., Tsopanos S., Theobald M.D., Nurick G.N., Langdon G.S. (2010). The quasi-static response of steel lattice structures. J. Sandw. Struct. Mater..

[13] Shen Y., Cantwell W.J., Mines R., Ushijima K. (2012). The properties of lattice structures manufactured using Selective Laser Melting. Adv. Mater. Res..

[14] Dotcheva M., Millward H., Thomas D. Investigation of Rapid Manufactured Cellular Structures for Injection Moulding Tool. Proceedings of the 6th CIRP International Conference on Intelligent Computation in Manufacturing Engineering.

[15] Contuzzi N., Campanelli S.L., Casavola C., Lamberti L. (2013). Manufacturing and characterization of 18Ni Marage 300 Lattice components by Selective Laser Melting. Materials.

[16] Ramirez D.A., Murr L.E., Li S.J., Tian Y.X., Martinez E., Machado B.I., Martinez J.L., Gaytan S.M., Medina F., Wicker R.B. (2011). Open-cellular copper structures fabricated by additive manufacturing using electron beam melting. Mater. Sci. Eng. A.

[17] Hasan R., Mines R., Shen E., Tsopanos S., Cantwell W., Brooks W., Sutcliffe C. (2010). Comparison of the drop weight impact performance of sandwich panels with aluminium honeycomb and titanium alloy micro-lattice cores. Appl. Mech. Mater..

[18] Hasan R., Mines R., Shen E., Tsopanos S., Cantwell W. (2011). Comparison on compressive behaviour of aluminium honeycomb and titanium alloy micro lattice blocks. Key Eng. Mater..

[19] Polmear I.J. (2006). Light Alloys: From Traditional Alloys to Nanocrystals.

[20] Wu X. (2006). Review of alloy and process development of TiAl alloys. Intermetallics.

[21] Yadroitsev I., Krakhmalev P., Yadroitsava I. (2014). Selective laser melting of Ti6Al4V alloy for biomedical applications: Temperature monitoring and microstructural evolution. J. Alloy. Compd..

[22] Hunt J.A., Callaghan J.T., Sutcliffe C.J., Morgan R.H., Halford B., Black R.A. (2005). The design and production of Co–Cr alloy implants with controlled surface topography by CAD–CAM method and their effects on osseointegration. Biomaterials.

[23] Murr L.E., Gaytan S.M., Martinez E., Medina F., Wicker R.B. (2012). Next generation orthopedic implants by additive manufacturing using electron beam melting. Int. J. Biomater..

[24] Parthasarathy J., Starly B., Raman S., Christensen A. (2010). Mechanical evaluation of porous titanium (Ti-6Al-4V) structures with Electron Beam Melting (EBM). J. Mech. Behav. Biomed. Mater..

[25] Harrysson O., Cansizoglu O., Marcellin-Little D., Cormier D., West H. (2008). Direct metal fabrication of titanium implants with tailored materials and mechanical properties using electron beam melting technology. Mater. Sci. Eng. C.

[26] Hasan R., Mines R., Fox P. (2011). Characterization of selectively laser melted Ti-6Al-4V micro-lattice struts. Procedia Eng..

[27] Bandyopadhyay A., Espana F., Balla V.K., Bose S., Ohgami Y., Davies N.M. (2010). Influence of porosity on mechanical properties and *in vivo* response of Ti6Al4V implants. Acta Biomater..

[28] Cardaropoli F., Alfieri V., Caiazzo F., Sergi V. (2012). Manufacturing of porous biomaterials for dental implant applications through Selective Laser Melting. Adv. Mater. Res..

[29] Li X., Wang C., Zhang W., Li Y. (2010). Fabrication and compressive properties of Ti6Al4V implant with honeycomb-like structure for biomedical applications. Rapid Prototyp. J..

[30] Lin C.Y., Wirtz T., LaMarca F., Hollister S.J. (2007). Structural and mechanical evaluations of a topology optimized titanium interbody fusion cage fabricated by selective laser melting process. J. Biomed. Mater. Res. Part A.

[31] Habijan T., Haberland C., Meier H., Frenzel J., Wittsiepe J., Wuwer C., Greulich C., Schildhauer T.A., Koller M. (2013). The biocompatibility of dense and porous nickel-titanium produced by selective laser melting. Mater. Sci. Eng. C.

[32] Emmelmann C., Scheinemann P., Munsch M., Seyda V. (2011). Laser additive manufacturing of modified implant surfaces with osseointegrative characteristics. Phys. Procedia.

[33] Cardaropoli F., Alfieri V., Caiazzo F., Sergi V. (2012). Dimensional analysis for the definition of the influence of process parameters in selective laser melting of Ti–6Al–4V alloy. Proc. Inst. Mech. Eng. Part B: J. Eng. Manuf..

[34] Wong M., Eulenberger J., Schenk R., Hunziker E. (1995). Effect of surface topology on the osseointegration of implant materials in trabecular bone. J. Biomed. Mater. Res..

[35] (2000). ISO 4288. Geometrical Product Specifications (GPS) – Surface Texture: Profile Method – Rules And Procedures For The Assessment Of Surface Texture.

[36] (2004). Annual Book of ASTM Standards. Medical Devices and Services.

[37] Kooistra G.W., Deshpande V.S., Wadley H.N.G. (2004). Compressive behaviour of age hardenable tetrahedral lattice truss structures made from aluminium. Acta Mater..

[38] Ashby M.F., Evans A.G., Fleck N.A., Gibson L.J., Hutchinson J.W., Wadley H.N.G. (2001). Metal Foams: A Design Guide.

[39] Douglas T., Queheillalt D.T., Wadley H.N.G. (2005). Pyramidal lattice truss structures with hollow trusses. Mater. Sci. Eng. A.

[40] Montgomery D.C., Runger G.C. (2003). Applied Statistics and Probability for Engineers.

[41] Cansizoglu O., Harrysson O., West H., Cormier D., Mahale T. (2008). Applications of structural optimization in direct metal fabrication. Rapid Prototyp. J..

[42] Brooks W., Sutcliffe C., Cantwell W., Fox P., Todd J., Mines R. Rapid Design and Manufacture of Ultralight Cellular Materials. the Proceedings of the Solid Freeform Fabrication.

[43] (2004). ASTM F1472. Standard Specification for Wrought Titanium-6Aluminum-4Vanadium Alloy for Surgical Implant Applications.

[44] Lu L., Fuh J.Y.H., Wong Y.S. (2001). Laser-Induced Materials and Processes for Rapid Prototyping.

[45] Badrossamay M., Childs T.H.C. (2007). Further studies in selective laser melting of stainless and tool steel powders. Int. J. Mach. Tools Manuf..

[46] Traini T., Mangano C., Sammons R.L., Mangano F., Macchi A., Piattelli A. (2008). Direct laser metal sintering as a new approach to fabrication of an isoelastic functionally graded material for manufacture of porous titanium dental implants. Dent. Mater..

[47] (2005). EN ISO 6507-1. Metallic Materials—Vickers Hardness Test.

